# Response to "Anatomic Twist to a Straightforward Ablation"

**DOI:** 10.1016/s0972-6292(16)30695-7

**Published:** 2013-11-15

**Authors:** David Chase, A Devi, Bobby John

**Affiliations:** Department of Cardiac Pacing and Electrophysiology, Christian Medical College Hospital, Vellore, Tamilnadu, India, PIN 632004

**Keywords:** Ablation, Anatomic Twist

We have read with interest the article titled 'Anatomic Twist to a Straightforward Ablation' authored by Mandeep Singh Randhawa et al [1]. A detailed report of a commendable and unique successful Atrio-Ventricular junction ablation for rapid ventricular-rate control in a patient with Atrial Fibrillation in the setting of left atrial isomerism, common atrium and an interrupted infra-hepatic Inferior Venacava draining via an Azygos venous connection into the Superior venacava is made.

An alternative venous approach could have been a right internal jugular venous one, failing which, a retrograde aortic approach [2] is also feasible providing of course that attention is paid to the coagulation parameters (for example, if the patient is receiving some form of anticoagulation therapy), given that an arterial access would be required. The authors would probably have considered either of these alternative approaches first, as they should, in order to minimize the risk of damage to the Azygos vein or to it's patency due to actual injury or due to thrombus formation / dissection / hematoma causing extrinsic compression in the worst case scenario (that inadvertent ligation of the Azygos vein could cause death is well-known). The Schwartz right long sheath which has been used for facilitating the ablation is a stiff sheath and anything more than a smooth gradual curve could risk kinking the sheath (making catheter manipulations difficult), or else accordioning it (risk of Azygos vein injury or to it's tributaries during attempts to advance or withdraw the damaged sheath), not to mention the risk of shearing the sheath. Acute angulation stress to the sheath and maximum risk of injury would have been encountered at the level of the inferior venacaval interruption where it connects with the Azygos vein, and where the Azygos vein joins the superior venacava (no images indicating the connections and course inferiorly are available). In the setting of an interrupted inferior venacava, before other less-experienced operators attempt this kind of an approach, adequate thought needs to be given to ruling out less cumbersome options in view of the risks involved.
